# Photoelectrochemical Properties of Annealed Anodic TiO_2_ Layers Covered with CuO_x_

**DOI:** 10.3390/molecules27154789

**Published:** 2022-07-26

**Authors:** Karolina Syrek, Monika Sołtys-Mróz, Kinga Pawlik, Magdalena Gurgul, Grzegorz D. Sulka

**Affiliations:** Department of Physical Chemistry & Electrochemistry, Faculty of Chemistry, Jagiellonian University, Gronostajowa 2, 30387 Krakow, Poland; soltys@chemia.uj.edu.pl (M.S.-M.); kinga.a.pawlik@student.uj.edu.pl (K.P.); gurgulm@chemia.uj.edu.pl (M.G.); sulka@chemia.uj.edu.pl (G.D.S.)

**Keywords:** titanium oxide, copper oxide, anodization, impregnation, photoelectrochemical activity

## Abstract

In this work, we present a systematic study on the influence of Cu^2+^ ion concentration in the impregnation solution on the morphology, structure, optical, semiconducting, and photoelectrochemical properties of anodic CuO_x_-TiO_2_ materials. Studied materials were prepared by immersion in solutions with different concentrations of (CH_3_COO)_2_Cu and subjected to air-annealing at 400 °C, 500 °C, or 600 °C for 2 h. The complex characterization of all studied samples was performed using scanning electron microscopy (SEM), energy dispersive spectroscopy (EDS), X-ray diffraction (XRD), reflectance measurements, Mott–Schottky analyses, and photocurrent measurements. It was found that band gap engineering based on coupling CuO with TiO_2_ (E_g_~3.3 eV) is an effective strategy to increase the absorption in visible light due to band gap narrowing (CuO_x_-TiO_2_ materials had E_g_~2.4 eV). Although the photoactivity of CuO-TiO_2_ materials decreased in the UV range due to the deposition of CuO on the TiO_2_ surface, in the Vis range increased up to 600 nm at the same time.

## 1. Introduction

Photoelectrochemical water splitting is considered a promising method for the generation of clean energy. However, there are still many problems connected with this topic, such as the low efficiency of systems and their limited usability for ultraviolet radiation only. Semiconducting nanomaterials seem to be very attractive for this type of application, especially due to their unique geometry and resulting properties. Anodic titanium dioxide nanotubes are often used as photoanodes in the unchanged or modified form with metals, oxides, and other semiconductors [1,2,3].

A very attractive strategy investigated via a variety of synthesis methods [4,5,6,7,8,9,10,11] is to couple a wide band gap material with copper oxides since both of them (Cu_2_O and CuO) have a narrow band gap (~2 eV) [12], which would be beneficial for widening the absorption spectra of TiO_2_-based materials into the visible region. Over the years, much attention was devoted to the deposition of copper or its oxides on anodic TiO_2_ nanotubes and studying their properties. Table 1 presents examples of copper-modified TiO_2_ nanotubes synthesized by different methods, material compositions, and characteristics. As can be seen, the optical properties of CuO_x_-TiO_2_ materials are enhanced and the absorption edge position is observed in the visible range at around 650 nm, which is beneficial for photochemical processes under solar illumination. The application of CuO_x_-TiO_2_ in the photoelectrochemical water splitting experiments requires considering other copper oxide properties, such as conduction and valence band alignment and its conductivity. Anatase or rutile TiO_2_ structures are known for their large band gaps of 3.2 eV and 3.0 eV, respectively, and the fact that they can operate only under UV light to excite an electron from the valence band to the conduction band [13]. Therefore, suitable band gap engineering is necessary to utilize visible light for excitation. Both copper oxides ensure the narrowed band gap, and those p-type semiconductors are perfect candidates for creating a junction with an n-type TiO_2_ for enhanced photoelectrochemical properties [14]. Moreover, it is well known that thermal treatment is often necessary to convert amorphous anodic oxides to photoactive structures and has a significant influence on the morphology, crystallinity, and semiconductor properties. For instance, Masudy-Panah et al. [15] reported that the crystallinity and grain size of the CuO thin films are significantly influenced by the annealing temperature. It was shown that higher heat treatment conditions cause a decrease in the grain boundary area over a given distance and enhance photocatalytic water splitting due to a reduction in grain boundary scattering and charge carriers recombination rate.

In this work, we present a systematic study focusing on the influence of Cu^2+^ ion concentration in the impregnation solution on the morphology, structure, optical, semiconducting, and photoelectrochemical properties of anodic CuO_x_-TiO_2_ materials. Studied materials were prepared by immersion in solutions with different concentrations of (CH_3_COO)_2_Cu (10–100 mM) and subjected to air-annealing at 400 °C, 500 °C, or 600 °C for 2 h. A complex characterization of all studied samples was performed using scanning electron microscopy (SEM), energy dispersive spectroscopy (EDS), X-ray diffraction (XRD), reflectance measurements, Mott–Schottky analysis, and photocurrent measurements under monochromatic and solar radiation.

## 2. Materials and Methods

Titanium foil (95.5% purity, thickness 0.25 mm, Alfa Aesar) was polished electrochemically and chemically before electrochemical oxidation. The electrochemical polishing was conducted in a mixture (60 : 15 : 25 in volume) containing acetic acid (98 wt.%), sulfuric acid (98 wt.%), and hydrofluoric acid (40 wt.%) at a constant temperature (~10 °C) and current density (1.4 A∙cm^−2^) for 1 min. Afterward, the chemical polishing was performed by immersion of Ti samples into a mixture of hydrofluoric acid (40 wt.%) and nitric acid (65 wt.%) (1:3 in volume) for 10 s. Then, Ti samples were rinsed with water and ethanol, and dried in the stream of hot air. Nanoporous anodic titanium oxide layers were synthesized in an ethylene glycol-based solution containing NH_4_F (0.38 wt.%) and H_2_O (1.79 wt.%) [16]. Three-step electrolysis was carried out at the constant voltage of 40 V at 20 °C. The first and second anodizing steps were performed for 3 h. After each step, an adhesive tape was used for the mechanical removal of grown oxide layers. The third anodizing step was carried out for 10 min in a freshly prepared electrolyte. During each step, a constant stirring rate of 200 rpm was provided. As-received anodic TiO_2_ layers were soaked in copper acetate solutions (10, 25, 50, and 100 mM, pH~6.2). Each sample was immersed for 10 min and then dried at 80 °C for 20 min. The described procedure constituted one full impregnation cycle, which was repeated five times. Afterward, obtained materials were annealed in air at 400 °C, 500 °C, and 600 °C for 2 h using a muffle furnace (FCF 5SHM Z, Czylok) with a heating rate of 2 °C·min^−1^.

The morphology and chemical composition of synthesized materials were characterized using a field emission scanning electron microscope (FE-SEM/EDS, Hitachi S-4700 with a Noran System 7, Tokyo, Japan). The morphology of non-modified anodic TiO_2_ layers obtained at different temperatures with corresponding oxide thicknesses are presented in Figure S1 (Supplementary Materials). The phase composition was determined by using a Rigaku Mini Flex II (Rigaku, Tokyo, Japan) diffractometer with Cu Kα radiation (1.54060 Å) at the 2θ range of 20–60°. UV-Vis diffuse reflectance spectra were measured using a Lambda 750S spectrophotometer (PerkinElmer, Waltham, MA, USA) equipped with an integrating sphere.

Electrochemical and photoelectrochemical measurements were performed in a three-electrode system, where a saturated calomel electrode (SCE), platinum foil, and CuO_x_-TiO_2_ samples were used as a reference, counter, and working electrodes, respectively. The semiconducting properties of modified anodic TiO_2_ layers were studied based on Mott–Schottky analyses performed in the dark, at the constant frequency of 200, 500, and 1000 Hz in a 0.1 M KNO_3_ solution using a Gamry Instrument Reference 3000 potentiostat (Gamry Instruments, Warminster, PA, USA). Photoelectrochemical tests were carried out using a photoelectric spectrometer (Instytut Fotonowy, Kraków, Poland) equipped with a 150 W xenon arc lamp in a Teflon cell with a quartz window. The photocurrent vs. time curves were recorded at 1 V vs. SCE under solar or monochromatic light. A pulse illumination in the range of 300–600 nm with a 10 nm wavelength step and 10 s light and 10 s dark cycles were used. Solar illumination experiments were carried out using AM 1.5 G standard sunlight filter and a xenon light source 150 W (Instytut Fotonowy, Kraków, Poland) combined with PalmSens4 (PalmSens BV, Houten, The Netherlands) potentiostat.

## 3. Results

### 3.1. Morphology and Composition of CuO_x_-TiO_2_ Materials

**Figure 1:**
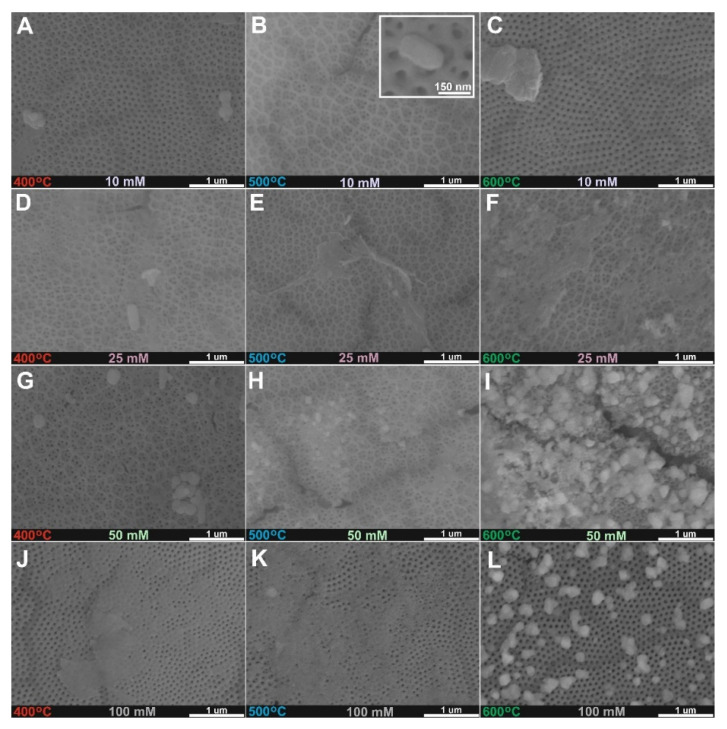
FE-SEM top views of anodic TiO_2_ samples annealed at 400 °C (**A**,**D**,**G**,**J**), 500 °C (**B**,**E**,**H**,**K**), and 600 °C (**C**,**F**,**I**,**L**) after impregnation in 10 mM (**A**–**C**), 25 Mm (**D**–**F**), 50 mM (**G**–**I**), and 100 mM (**J**–**L**) (CH_3_COO)_2_Cu solutions.

Anodic TiO_2_ layers were impregnated with 10–100 mM copper acetate solutions and annealed at 400 °C, 500 °C, and 600 °C for 2 h in the air to obtain CuO_x_ crystals on the top of nanoporous layers as it is presented in Figure 1. At the lower concentration of the solution (10 mM Cu^2+^, Figure 1A–C), a porous TiO_2_ surface is exposed and rather small crystals can be found. Similar morphology was found for samples after treatment in the solution with over two times higher concentrations (25 mM Cu^2+^, Figure 1D–F). For the higher concentration used (50 and 100 mM Cu^2+^ Figure 1G–L), the surface is partially, or totally covered by the deposited CuO_x_. Moreover, heat treatment at 600 °C enables the better formation of CuO_x_ crystals on the surface, which is best presented in Figure 1L. Similar heterogeneous morphology was found for anodic TiO_2_-Fe_2_O_3_ materials synthesized by the anodization-impregnation-annealing procedure [17,18].

**Figure 2:**
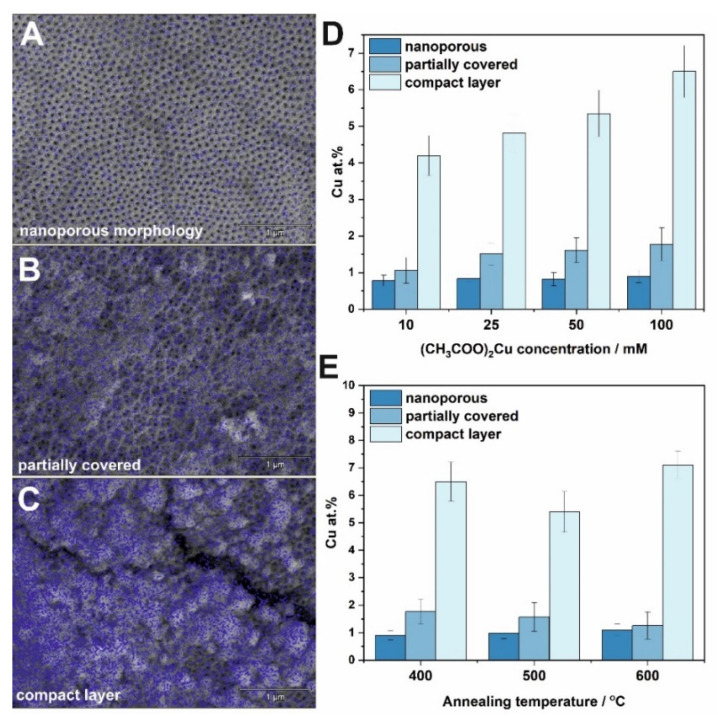
EDS maps showing copper distribution over TiO_2_ surface with different morphologies: nanoporous (**A**), partially covered (**B**), and a compact layer (**C**). The estimated atomic content of copper in different parts of materials as a function of copper acetate concentration (**D**), and annealing temperature (**E**) for samples impregnated in a 100 mM (CH_3_COO)_2_Cu solution.

To investigate the changes in copper content and distribution over the TiO_2_ surface, EDS mapping was performed in three different parts of each sample for three different morphology types (i) nanoporous (Figure 2A), (ii) partially covered with CuO_x_ (Figure 2B), and (iii) a compact layer (Figure 2C). The average atomic percent of copper detected for different impregnation concentrations of 10–100 mM and annealed at 400 °C are presented in Figure 2D. As can be seen, copper distribution over a porous surface is the most homogenous, which is indicated by blue marks in SEM microphotographs presented in Figure 2A, and dark blue bars in Figure 2D indicating the Cu content of ~0.9 at.% regardless of the annealing temperature. By comparing the copper total amount in partially covered areas of the samples, it can be concluded that the content is very similar and does not depend on the concentration of the impregnation solution, however, such a relationship can be seen for sites with a compact morphology (Figure 2D). The effect of the annealing temperature was tested at the highest concentration of the impregnation solution (100 mM Cu^2+^) in order to minimize the determination errors. In addition, as expected, the copper content for each type of tested morphology is very comparable (Figure 2E).

**Figure 3:**
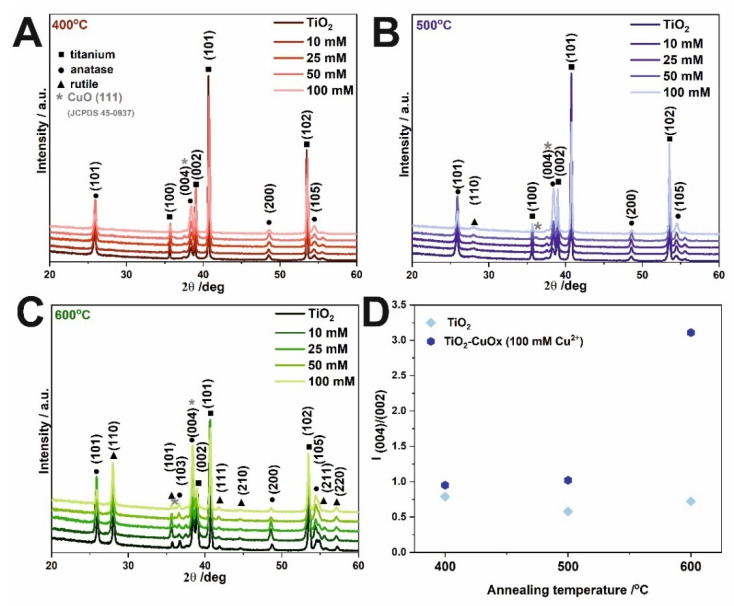
XRD patterns of TiO_2_ samples impregnated in (CH_3_COO)_2_Cu and annealed at 400 °C (**A**), 500 °C (**B**), and 600 °C (**C**). Estimated reflection (004):(002) ratio for unmodified and modified materials obtained as a result of impregnation in a 100 Mm solution and annealed at different temperatures (**D**).

The diffractograms obtained for samples unmodified and modified with Cu^2+^ ions and annealed at 400 °C, 500 °C, and 600 °C are presented in Figure 3A–C, respectively. Characteristic signals from the metallic substrate (JCPDS card no. 05-0682), anatase (JCPDS card no. 21-1272), rutile (JCPDS card no. 21-1276), and copper (II) oxide (JCPDS 05-0667) are marked. The results show that the crystal structure of the modified and non-modified TiO_2_ layers is very similar. Reflections from titanium (100), (002), (101), (102), (110), (103), (112), and (201) planes, and anatase (101), (004), (200), (105), and (116) planes were detected. At the 2Θ ~38–39°, the change in the intensity of the anatase plane (004) was observed. The reflection ratios of (004):(002) planes were calculated for bare and copper-modified samples annealed at different temperatures for materials impregnated with 100 mM (CH_3_COO)_2_Cu and presented in Figure 3D. As can be seen, results indicate the increase in the intensity of the (004) plane, which can be ascribed to the anatase plane, however, the maximum overlap with the CuO (111) plane. Moreover, Wojcieszak et al. [19] studied the effect of annealing temperature on the properties of copper oxide thin films and showed that copper (I) oxide transformation into CuO started at 300 °C, while the thin film annealed at 350 °C consisted of a single CuO phase. It is widely recognized that anatase to rutile transformation occurs above 400 °C, and this structure is indicated by planes (110), (101), (111), (210), (211), and (220) in Figure 3B,C.

### 3.2. Optical Properties

**Figure 4:**
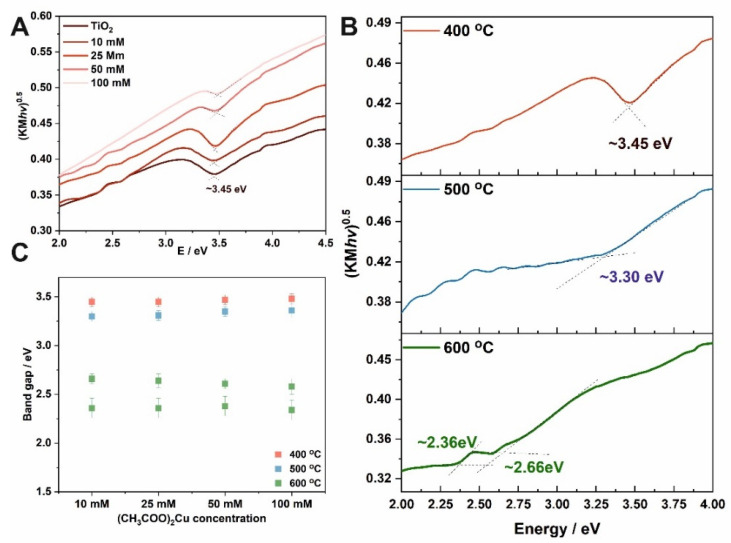
Tauc plots for TiO_2_-CuO_x_ materials obtained in solutions with different (CH_3_COO)_2_Cu concentrations and annealed at 400 °C (**A**). Tauc plots for TiO_2_ layers impregnated in a 10 mM (CH_3_COO)_2_Cu and annealed at different temperatures (**B**). Estimated optical band gap energies as a function of (CH_3_COO)_2_Cu concentration used for impregnation (**C**).

The optical properties of the tested materials were performed using UV-Vis diffuse reflectance spectroscopy. Band gap energies were determined from the obtained spectra using Equation (1):

(1) $${\left(\alpha \cdot h\nu \right)}^{1/\gamma }=B\cdot \left(h\nu -E_{g}\right)$$

where: α is the absorption coefficient; *h* is the Planck constant; *ν* is the frequency of the photon; γ is a constant, which takes a value of 2 for indirect and ½ for direct transition; B is a constant; *E_g_* is the band gap energy [20]. The DRS spectra were transformed using the Kubelka–Munk function given by (2):

(2) $$F\left(R_{\infty }\right)=\frac{{\left(1-R_{\infty }\right)}^{2}}{2R_{\infty }}$$

where: *R_∞_* is reflectance, *F*(*R_∞_*) is the Kubelka–Munk function. Band gaps of the tested materials were determined from Tauc plots by determining the intersection point of two linear segments of the function, as shown in Figure 4A,B. Materials obtained by impregnation in solutions with different concentrations and heated at 400 °C have a band gap of ~ 3.45 eV. Analyzing the changes in the band gap for higher annealing temperatures, it can be seen that band gap narrowing takes place (Figure 4B), and for 600 °C a second band gap can be observed. Moreover, Cu^2+^ ions may form sub-band states in the band gap of TiO_2_ [21]. Along with Cu^2+^, oxygen defects band states are also formed in the band gap. In pure TiO_2_, the electronic transition occurs directly from the valence to the conduction band. However, while considering the Cu doping, the electrons are not directly excited to the conduction band, since the unoccupied Cu^2+^ s-d states and oxygen vacancies may capture the electrons, which results in a reduction in the effective band gap of TiO_2_ [21,22].

### 3.3. Semiconducting Properties of CuO_x_-TiO_2_ Materials

**Figure 5:**
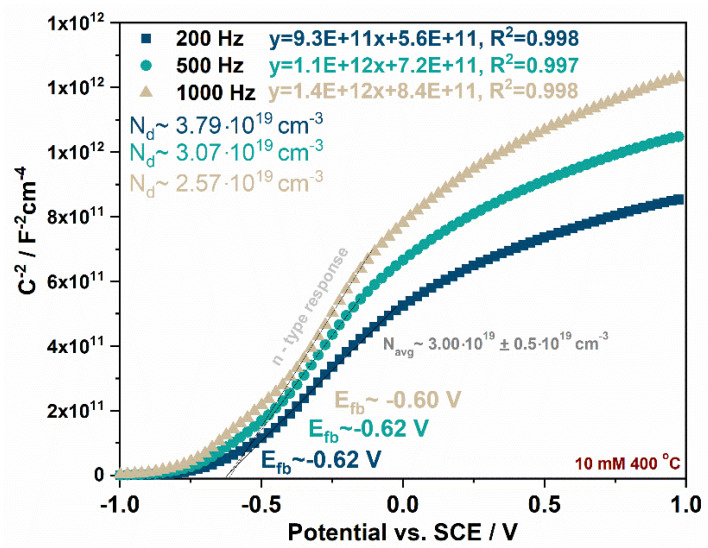
Mott–Schottky plots recorded for samples obtained by impregnation in a 10 mM Cu^2+^ solution and annealed at 400 °C at different frequencies with calculated the flat band potential and donor density for each curve.

In order to study the semiconducting behavior of synthesized materials, the Mott–Schottky analysis was performed. The relationship between semiconductor–electrolyte interfacial capacitance and potential follows the Formula (3) [16,23]:

(3) $$C_{SC}^{-2}=\left(\frac{2}{\varepsilon \varepsilon_{0}qN_{d}}\right)\left(E-E_{fb}-\frac{kT}{q}\right)$$

where: C_sc_ is the capacitance of the space charge region (F·cm^−2^), N_d_ is the donor density (cm^−3^), ɛ is the dielectric constant of TiO_2_ (100) [16], ɛ_0_ is the permittivity of free space (8.85 × 10^−14^ F·cm^−1^), q is the electron charge (1.602 × 10^−19^ C), E is the applied potential (V), T is the absolute temperature (K), and k is the Boltzmann constant (1.38 × 10^−23^ J·K^−1^). The positive slope of the linear dependence of C_sc_^−2^ on the potential indicates n-type semiconducting behavior, which is typical for anodic TiO_2_, while the negative slope suggests p-type behavior, which is connected to the presence of CuO_x_ in the studied materials. The flat band potential for each material was estimated at 200, 500, and 1000 Hz. The Mott–Schottky plots recorded for the sample obtained by impregnation in a 10 mM Cu^2+^ solution and annealed at 400 °C measured at different frequencies are presented in Figure 5. The flat band potentials vs. SCE (pH~5.9) were estimated at studied frequencies, and the results are gathered in Table 2. Moreover, for each plot, the donor density was calculated and averaged for each sample, as shown in Figure 5. It is common for nanocrystalline porous electrodes to observe some differences in E_fb_ over the studied frequency range due to electrode porosity and the metal components, which form the conduction band [16,23,24]. Moreover, in some cases, the Mott–Schottky plot may be characterized by three specific regions described from negative to positive potentials that correspond to the depletion layer model for semiconductor electrodes. The first linear region corresponds to lower band bending and smaller penetration depth, so E_fb_ values may be closer to a real value, but calculating N_d_ from this slope is affected by the surface morphology and can be different from the donor density in the bulk. The second linear part of the curve can be observed, which is preceded by a plateau region related to the presence of surface states [23,25].

**Figure 6:**
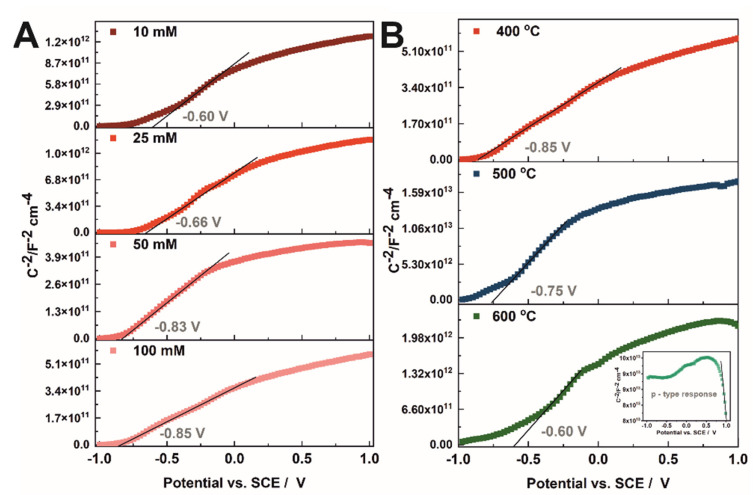
Mott–Schottky plots recorded for samples obtained by impregnation in 10–100 mM Cu^2+^ solutions and annealed at 400 °C (**A**) and impregnated in 100 mM Cu^2+^ and annealed at 400–600 °C (**B**).

With increasing the concentration of copper acetate in the solution used for the preparation of CuO_x_-TiO_2_ materials, the conduction band edge shifts towards more negative potentials, and E_fb_ of −0.60, −0.66, −0.83, and −0.85 V vs. SCE were determined at 1 kHz for the (CH_3_COO)_2_Cu solution of 10, 25, 50, and 100 mM (Figure 6A and Table 2), respectively. This effect is in agreement with the data reported for FeO_x_-TiO_2_ materials, where increasing the concentration of ferric chloride solution induces a shift of the conduction band edge to negative values [17]. As can be seen, the flat band potential of CuO_x_-TiO_2_ annealed at 400 °C is more negative (−0.85 V vs. SCE) than those obtained for the samples annealed at higher temperatures (−0.75 V and −0.60 V vs. SCE for the sample annealed at 500 °C and 600 °C, respectively). This observation is in opposition to flat band potential changes over the annealing temperature of anodic TiO_2_ [16] and is directly connected to the introduction of the CuO phase. As shown earlier, the morphology of the materials obtained is heterogeneous, and probably if larger aggregates of CuO are produced on the material surface, the character of the sample changes to the p-type conductivity, as it is presented in Figure 6B inset. Moreover, the donor density increases from 3.00 × 10^19^ cm^−3^ to 4.55 × 10^19^ (±0.2 × 10^19^) cm^−3^ with increasing the Cu^2+^ concentration in the impregnation solution for the annealing temperature of 400 °C. When the annealing temperature changes to 600 °C, an N_d_ decrease to 1.50 × 10^19^ (±0.2 × 10^19^) cm^−3^ is observed.

### 3.4. Photoelectrochemical Properties of CuO_x_-TiO_2_ Photoelectrodes

**Figure 7:**
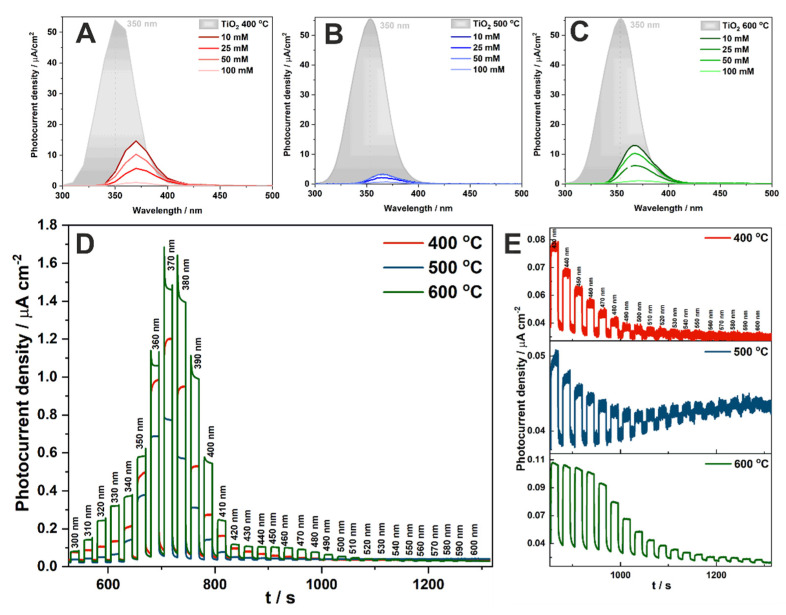
Photocurrent density vs. wavelength curves obtained for anodic TiO_2_ and CuO_x_-TiO_2_ materials annealed at 400 °C (**A**), 500 °C (**B**), and 600 °C (**C**) recorded at 1 V vs. SCE. Photocurrent density vs. time curves recorded at 1 V vs. SCE for CuO_x_-TiO_2_ materials impregnated with a 100 mM Cu^2+^ solution and heat-treated at different temperatures during its sequential illumination with the wavelength range of 300–600 nm (**D**) and 430–600 nm (**E**).

Photocurrent density vs. wavelength curves for anodic TiO_2_ and CuO_x_-TiO_2_ materials annealed at 400 °C, 500 °C, and 600 °C were recorded at 1 V vs. SCE and are presented in Figure 7A–C, respectively. Dark grey spectra represent the photoresponse of anodic TiO_2_ annealed at each temperature. It is visible, that impregnation in the Cu^2+^ solution changed the material properties. Due to the deposition of CuO on the TiO_2_ surface, the photoactivity in the UV range decreased, while the activity range of CuO-TiO_2_ materials was extended up to 600 nm (Figure 7D,E). A similar tendency was observed in other work, for materials impregnated with various FeCl_3_ concentrations [17]. Photocurrent spectra have been extended for materials impregnated in the solution with the highest concentration of Cu^2+^ (100 mM), and their comparison for different temperatures is shown in Figure 7D. It is clearly seen that higher photocurrent densities are generated for materials annealed at 600 °C, and the current generated in the visible light range is better established (Figure 7E) than in the other cases. This is probably related to the changes in the crystallinity of the CuO phase with the annealing temperature and formation of the n–p junction (as indicated by the Mott–Schottky measurements for the highest temperature treatment, please see inset in Figure 6B).

**Figure 8:**
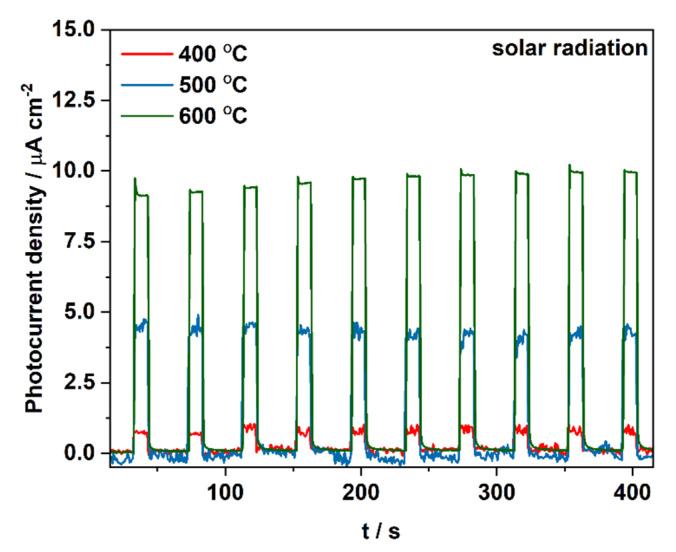
Photocurrent density registered during sequential illumination with white light using AirMass1.5 filter at 1 V vs. SCE for CuO_x_-TiO_2_ materials impregnated with a 100 mM Cu^2+^ solution and heat treated at different temperatures.

At the final stage, materials impregnated with a 100 mM Cu^2+^ solution and heat-treated at different temperatures were tested under solar illumination in order to evaluate their overall photoelectrochemical performance (Figure 8). As expected, the material annealed at 600 °C generates twice higher photocurrent than the material heat-treated at 500 °C and over ten times higher than the sample obtained at 400 °C. This observation is in the line with changes in the flat band potential of tested materials.

## 4. Conclusions

The morphology of anodic TiO_2_ samples immersed in a copper acetate solution of different concentrations and annealed at 400–600 °C was investigated. It was found that obtained samples were heterogeneous and three different types of morphology with different copper contents are observed: (i) nanoporous, (ii) partially covered with CuO_x_, and (iii) a compact layer). The XRD analysis revealed the presence of anatase, rutile (with different ratios depending on the applied annealing temperature), and CuO phases. The bang gap energies were studied, and no differences in the E_g_ values were observed with increasing the concentration of copper (II) ions in the impregnating solution, while with increasing the annealing temperature, a gradual narrowing of the band gap energy was observed (from 3.3 eV to 2.4 eV for the materials annealed at 400 °C and 600 °C, respectively). Due to the deposition of CuO on the TiO_2_ surface, the photoactivity in the UV range decreased (gradually with increasing the Cu^2+^ ions concentration in the solution used for impregnation), while the photoactivity of CuO-TiO_2_ materials was extended up to 600 nm. Clearly, the band gap engineering, based on coupling CuO with TiO_2_, is an effective strategy to increase the absorption of wide band gap semiconductor in visible light.

## Figures and Tables

**Table 1:** Different synthesis methods of copper-modified TiO_2_ nanotubes and their optical properties.

Method	Final Material	Optical Properties	Ref.
Electrochemical deposition	Cu_2_O-TiO_2_	Eg~2.17 eV	[4]
SILAR	Cu NPs-TiO_2_	absorption peak at ~570 nm	[5]
Electroplating	Cu-TiO_2_	-	[6]
Sonoelectrochemical	TiO_2_-Cu_2_O	enhanced absorption in the visible range	[7]
Electrodeposition followed by anodization	Cu_2_O-TiO_2_	absorption peak at ~620 nm	[8]
SILAR	CuO-TiO_2_	Eg~2.44 eV	[9]
In-situ anodization	CuO-TiO_2_	Eg~2.65 eV	[10]
Dip-coating followed by calcination	CuO/Cu_2_O/Cu-TiO_2_	adsorption edge at ~650 nm	[11]

**Table 2:** Flat band potential estimated at different frequencies (V vs. SCE).

Concentration of (CH_3_COO)_2_Cu	Annealing Temperature/°C	200 Hz	500 Hz	1000 Hz
10	400	−0.62	−0.62	−0.60
25	400	−0.70	−0.63	−0.66
50	400	−0.78	−0.76	−0.83
100	400	−0.80	−0.80	−0.85
100	500	−0.70	−0.74	−0.75
100	600	−0.66	−0.65	−0.60

## Data Availability

Not applicable.

## Supplementary Materials

The following supporting information can be downloaded at: https://www.mdpi.com/article/10.3390/molecules27154789/s1, Figure S1: Top and cross-sectional views of anodic TiO_2_ layers annealed at 400–600 °C.

## Sample Availability

Samples of the compounds are not available from the authors.
