# Supplementary material for: Photoelectrochemical Properties of Annealed Anodic TiO2 Layers Covered with CuOx
Source: Molecules. 2022 Jul 26;27(15):4789. doi: 10.3390/molecules27154789 (PMC9369738; doi:10.3390/molecules27154789)
Supplement: Supplementary file 1 [file molecules-27-04789-s001.zip › molecules-1800089-supplementary.pdf]

Supplementary Materials

Photoelectrochemical Properties of Annealed Anodic TiO_2 Layers Covered with CuO_x

Karolina Syrek *, Monika Sołtys-Mróz, Kinga Pawlik, Magdalena Gurgul and Grzegorz D. Sulka

Department of Physical Chemistry & Electrochemistry, Jagiellonian University, Faculty of Chemistry, Gronostajowa 2, 30387 Krakow, Poland

* Correspondence: syrek@chemia.uj.edu.pl

1. Morphology of TiO_2 layers

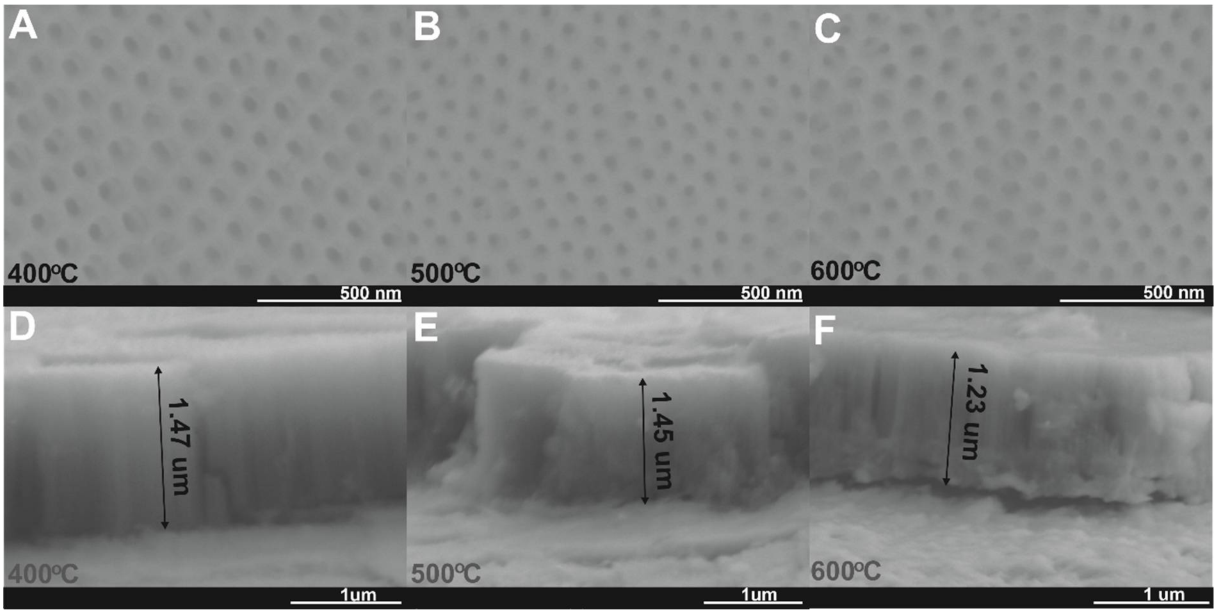

Figure S1. Top (A-C) and cross-sectional views of anodic TiO_2 layers annealed at 400 °C (A,D), 500 °C (B,E), and 600 °C (C,F).
